# 10-Year Paclitaxel Dose-Related Outcomes of Drug-Eluting Stents Treated Below the Knee in Patients with Chronic Limb-Threatening Ischemia (The PADI Trial)

**DOI:** 10.1007/s00270-020-02602-6

**Published:** 2020-07-28

**Authors:** Louise C. D. Konijn, Thijs Wakkie, Marlon I. Spreen, Pim A. de Jong, Lukas C. van Dijk, Jan J. Wever, Hugo T. C. Veger, Randolph G. Statius van Eps, Willem P. Th. M. Mali, Hendrik van Overhagen

**Affiliations:** 1 Department of Radiology, Haga Teaching Hospital, Leyweg 275, 2545CH/PO box 40551, The Hague, The Netherlands; 2 Department of Radiology, University Medical Center Utrecht and Utrecht University, Utrecht, The Netherlands; 3 Department of Vascular Surgery, Haga Teaching Hospital, Leyweg 275, 2545CH/PO box 40551, The Hague, The Netherlands

**Keywords:** Chronic limb-threatening ischemia, Below the knee, Drug-eluting stents, Paclitaxel, Mortality, Dose-related analysis

## Abstract

**Purpose:**

Recently, two meta-analyses concluded that there appears to be an increased risk of long-term mortality of paclitaxel-coated balloons and stents in the superficial femoral and popliteal artery, and paclitaxel-coated balloons below the knee. In this post hoc study of the PADI Trial, we investigated the long-term safety of first-generation paclitaxel-coated drug-eluting stents (DES) below the knee and the dose–mortality relationships of paclitaxel in patients with chronic limb-threatening ischemia (CLI).

**Materials and Methods:**

The PADI Trial compared paclitaxel-coated DES with percutaneous transluminal angioplasty with bail-out bare-metal stents (PTA ± BMS) in patients with CLI treated below the knee. Follow-up was extended to 10 years after the first inclusion, and survival analyses were performed. In addition, dose-related mortality and dose per patient weight-related mortality relations were examined.

**Results:**

A total of 140 limbs in 137 patients were included in the PADI Trial. Ten years after the first inclusion, 109/137 (79.6%) patients had died. There was no significant difference between mortality in the DES group compared with the PTA ± BMS group (Log-rank *p *value = 0.12). No specific dose-related mortality (HR 1.00, 95% CI 0.99–1.00, *p* = 0.99) or dose per weight mortality (HR 1.05, 95% CI 0.93–1.18, *p* = 0.46) relationships were identified in the Cox-proportional Hazard models or by Kaplan–Meier survival analyses.

**Conclusions:**

There is a poor 10-year survival in both paclitaxel-coated DES and PTA ± BMS in patients with CLI treated below the knee. No dose-related adverse effects of paclitaxel-coated DES were observed in our study of patients with CLI treated below the knee.

**Level of Evidence:**

The PADI Trial: level 1, randomized clinical trial

**Electronic supplementary material:**

The online version of this article (10.1007/s00270-020-02602-6) contains supplementary material, which is available to authorized users.

## Introduction

Drug-eluting stents (DES) were developed to improve patency with less in-stent restenosis caused by intimal hyperplasia, which is the most common constraint of bare-metal stents (BMS) [1–4]. DES were implemented in 2012 in patients with peripheral arterial disease (PAD). To compare the performance of paclitaxel-coated DES and standard treatment, percutaneous transluminal angioplasty with bail-out BMS (PTA ± BMS) below the knee (BTK) in patients with chronic limb-threatening ischemia (CLI), the Percutaneous transluminal Angioplasty versus Drug eluting stents for Infrapopliteal lesions (PADI) Trial was conducted [5]. This randomized clinical trial (RCT) showed a significantly better patency and a higher amputation-free and event-free survival at 5 years with paclitaxel-coated DES compared with the current reference treatment PTA ± BMS [6, 7]. These results are consistent with other studies that show lower restenosis rates in infrapopliteal lesions treated with DES in their follow-up [3, 8, 9].

Recently, two meta-analyses were published that concluded that there appears to be an increased risk of 5-year mortality of paclitaxel-coated drug-eluting balloons and DES in the superficial femoral and popliteal artery [10], and a significantly worse 1-year amputation-free survival in patients treated with DEB below the knee [11]. The vascular areas and devices used in the PADI study are not the same as in the published meta-analyzes, and the survival results are not consistent either.

The aim of this study was to extend the PADI Trial long-term follow-up to 10 years after the first inclusion and to evaluate the mortality to assess whether the use of paclitaxel-coated DES in our population is safe.

## Materials and Methods

### The PADI Trial

#### Study Approval

The medical ethical board of the participating centers approved the PADI Trial (Unique identifier number: NCT00471289), and written informed consent was obtained from all patients. The present study is a post hoc study of this RCT.

#### Patient Population

The PADI Trial was an investigator initiated prospective, multicenter RCT that compared the patency and clinical performance of paclitaxel-coated drug-eluting stainless-steel coronary stents (TAXUS Liberté stent system; Boston Scientific Corporation, Natick, United States) versus the current reference treatment with PTA ± BMS in patients with CLI treated infrapopliteal/BTK. Extensive information of the study protocol and mid- to long-term results can be found in previous publications [5–7, 12].

Briefly, between October 2007 and February 2013, patients with CLI who were treated BTK were included in three hospitals in the Netherlands. A total of 144 limbs in 141 patients were included. Randomization was performed per limb. Four patients were included for 2 limbs with 1 limb in each study arm. Four limbs/patients were excluded, 1 patient (1 limb) in the DES arm and in the PTA arm 3 patients (3 limbs), because of intermitted claudication (IC) (*n* = 1), renal failure without dialysis (*n* = 1), coagulation disorder (*n* = 1) or a too small vessel for treatment (*n* = 1). In total, 140 limbs in 137 patients were included for analysis of which 74 limbs in 73 patients for paclitaxel-coated DES and 66 for PTA ± BMS in 64 patients.

### Methods

In the PADI Trial, stenosis of the lesions had to be at least > 50%, the lesion length ≤ 90 mm and the arterial diameter between 2 to 6 mm. Bail-out BMS was used in case of flow limiting lesions, rest stenosis > 50% or occlusion after PTA.

For both the DES and the PTA ± BMS group, the stent lengths and stent diameters were noted in the case report form. Per patient, a maximum of three DES were placed and the total number of stents was recorded. Per DES, the paclitaxel dose was then calculated on the basis of its length and diameter, according to the TAXUS Liberté product information [13]. For an overview of the used DES sizes in this study, see supplemental Table 1.

The Taxus Liberté stents are manufactured from a 316L stainless steel stent. This stent is coated with a drug-polimer coating (consisting of a mix of paclitaxel and a Translute polymer carrier, directly applied on the stent). Paclitaxel is the active pharmaceutical ingredient, with the molecular formula of C47H51NO14. There is no topcoat layer or primer.

### Follow-up

Follow-up in the PADI Trial consisted of computed tomography (CT) angiography, duplex ultrasound and clinical assessment at 6 months. After that, patients were assessed annually for a period of 5 years after treatment by medical history, physical examination and duplex ultrasound of the treated limb. If patients were unavailable for follow-up, the information was gathered from general practitioners by phone or from digital medical records. The 5-year follow-up data have been published previously [7]. Regarding the follow-up data until April 2019 (10 years) for this current report, municipal basic records were checked for death and date of death.

### Statistical Analysis

Data analysis was carried out using SPSS version 24.0 for Windows (IBM Corporation, New York, United States). Baseline characteristics were assessed to describe the study population.

Continuous variables were calculated as means with standard deviations (SD). Categorical variables were presented as counts and percentages and tested with the Chi-squared tests. *p *values were tested two-sided; a *p *value of less than 0.05 was considered to be significant.

Survival of patients was assessed up to ten years after the first inclusion, and 10-year all-cause mortality for all patients was estimated using Kaplan–Meier plots. Significance was tested by Log-Rank (Mantel–Cox) tests.

The average dose of paclitaxel per stent, the total dose per patient and the dose per body weight ratio (μcg/kg) were assessed. Plotted in a stacked figure are the number of patients per dose (0–700 μcg). All Cox-proportional hazard models were performed for 10-year all-cause mortality. Cumulative dose was stratified per 150 μcg (0–149, 150–299, 300–449, 450–700 μcg) and univariate analyses and age- and sex-adjusted effects were calculated by hazard ratios with 95% confidence intervals (CI). In addition, separate univariate analyses were performed for paclitaxel-coated DES (dichotomous) and total dose per kilogram body weight (continuous). Multivariate modelling was performed included paclitaxel-coated DES and known risk factors of CLI: age, smoking, history of PAD, diabetes mellitus, previous stroke or transient ischemic attack, coronary arterial disease and the use of anticoagulant medication.

## Results

### Baseline Characteristics

At the time of treatment, mean age in the DES group was 74.2 ± 12.1 years and in the PTA ± BMS group 72.9 ± 11.9 years. 67.1% of the patients in the DES group were male. Overall, patients treated with DES were more likely to have a history of PAD than patients treated with PTA ± BMS (72.7% vs 55.4%, respectively, *p *value = 0.03). Patients treated with DES also had a higher percentage of previous amputation (34.8% vs 18.9%, respectively, *p *value = 0.03). Baseline characteristics are summarized in Table 1.

**Table 1:** Baseline characteristics total group (paclitaxel-DES and PTA ± BMS), total 140 limbs in 137 patients, of which 74 DES limbs in 73 patients and 66 PTA ± BMS limbs in 64 patients

	PTA ± BMS (*n* = 64)		Paclitaxel-DES (*n* = 73)		*p* value
	Mean ± SD	N(%)/min–max	Mean ± SD	*N* (%)/min–max	
Age (years)	72.9 ± 11.9	40–93	74.2 ± 12.1	40–98	
Sex (male)		47 (73.4%)		49 (67.1%)	0.64
Smoking status					
Smoker		17 (26.6%)		16 (21.9%)	0.64
Ex-smoker		12 (18.8%)		18 (24.7%)	
Diabetes mellitus		43 (67.2%)		44 (60.3%)	0.36
Previous stroke or TIA		13 (20.3%)		12 (16.4%)	0.60
Coronary artery disease		25 (39.1%)		27 (37%)	0.87
History of PAD		41(55.4%)		48(72.7%)	0.03*
Previous amputation		14(18.9%)		23(34.8%)	0.03*
On anticoagulant medication		58 (90.6%)		67 (91.8%)	0.58
Impaired renal function (eGFR < 30)		10 (15.6%)		10 (13.7%)	0.75
Rutherford at baseline					
4		8 (12.1%)		10 (13.5%)	0.83
5		46 (69.7%)		48 (64.9%)	
6		12 (18.2%)		16 (21.6%)	
Ankle-brachial index	0.81 ± 0.28	0.15-imm	0.89 ± 0.28	0.22-imm	

*PTA* percutaneous transluminal angioplasty, *BMS* bail-out bare-metal stents, *DES* drug-eluting stent, *TIA* transient ischemic attack, *PAD* peripheral arterial disease, *eGFR* electronic glomerular filtration rate (mL/min/1.73m^2^), *imm.* immeasurable high ankle-brachial index

*p value < 0.05

**Fig. 1:**
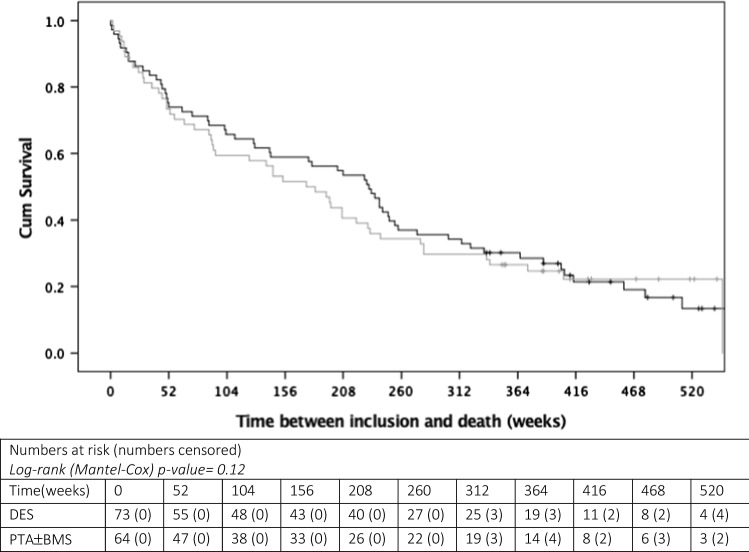
10-year Kaplan–Meier survival curves for DES (black line) versus PTA ± BMS (grey line)

Kaplan–Meier curves up to 10 years after the first inclusion are shown in Fig. 1. After 10 years, the majority patients had died 109/137 (79.6%). There was no significant difference between mortality in the DES and PTA ± BMS group, 59/73 (80.8%) and 50/64 (78.1%), respectively [Log-rank (Mantel–Cox) *p *value  = 0.12]. Mean survival was 236.7 weeks (95% CI 203.0 – 270.3 weeks) with a maximum follow-up of 520 weeks.

There was also no significant difference in mortality between the different Rutherford classes included; 4, 5 or 6 (Log-Rank (Mantel–Cox) *p* = 0.13). However, a non-significant difference in mortality between Rutherford 4 versus Rutherford 5 and 6 was noticeable. Rutherford 4 had a mean survival of 304.3 weeks (95% CI 208.6–400.0 weeks) versus Rutherford 5 with a mean survival of 199.9 (95% CI 164.0–235.7 weeks) and Rutherford 6 a mean survival of 191.04 (95% CI 129.3–252.8 weeks).

Interestingly, a significant Log-Rank (Mantel–Cox) value was found for impaired renal function; *p* = 0.04. Mean survival of patients with a renal function lower than 30 mL/min/1.73 m^2^ was 144.3 weeks (95% CI 70.9–217.6 weeks), while patients with a renal function higher than 30 mL/min/1.73 m^2^ had a significant longer mean survival of 226.3 weeks (95% CI 192.5–260.0 weeks). Corresponding Kaplan–Meier curves of different Rutherford classes and impaired renal function are shown in supplemental figures 1 and 2.

### Paclitaxel-Coated Drug-Eluting Stents and Dose

The average number of stents in the DES group was 1.81 ± 0.84. There were no significant differences in comorbidity per total number of stents placed per patient. In the DES group, the stent diameters ranged between 2 and 4 mm (mean 2.83 ± 0.40). Length of stents ranged between 16 and 38 mm (mean 29.74 ± 4.30). The paclitaxel dose per DES varied between 77.00 and 273.00μcg (mean 182.27 ± 39.05). See Table 2 for more detailed information.

**Table 2:** Stents, dose, weight and the relation of these parameters for patients treated with paclitaxel-coated DES in the PADI Trial

	Number (%)	Mean ± SD	Min–max
Stents			
Number of stents		1.81 ± 0.84	1.00–3.00
Diameter (mm)		2.83 ± 0.40	2.00–4.00
Length (mm)		29.74 ± 4.3	16–38.00
Dose per stent			
Total placed stents [numbers and dose (μcg)]			
1	30 (41.1%)	172.69 ± 40.87	116.00–266.00
2	29 (39.7%)	366.75 ± 80.30	213.00–532.00
3	14 (19.2%)	521.21 ± 79.15	361.00–645.00
Dose per gender and per weight			
Sex (numbers and dose (μcg))			
Male	49 (67.1%)	336.61 ± 158.87	116.00–645.00
Female	24 (32.9%)	273.74 ± 131.15	116.00–478.00
Weight (kg)		75.6 ± 12.9	47.0–122.0
Dose per patient (μcg)		315.03 ± 151.93	116.00–645.00
Dose/weight (μcg/kg)		4.30 ± 2.28	1.30–10.99

Per patient 3 stents maximum are used. Data are displayed as mean ± SD, minimum and maximum

**Fig. 2:**
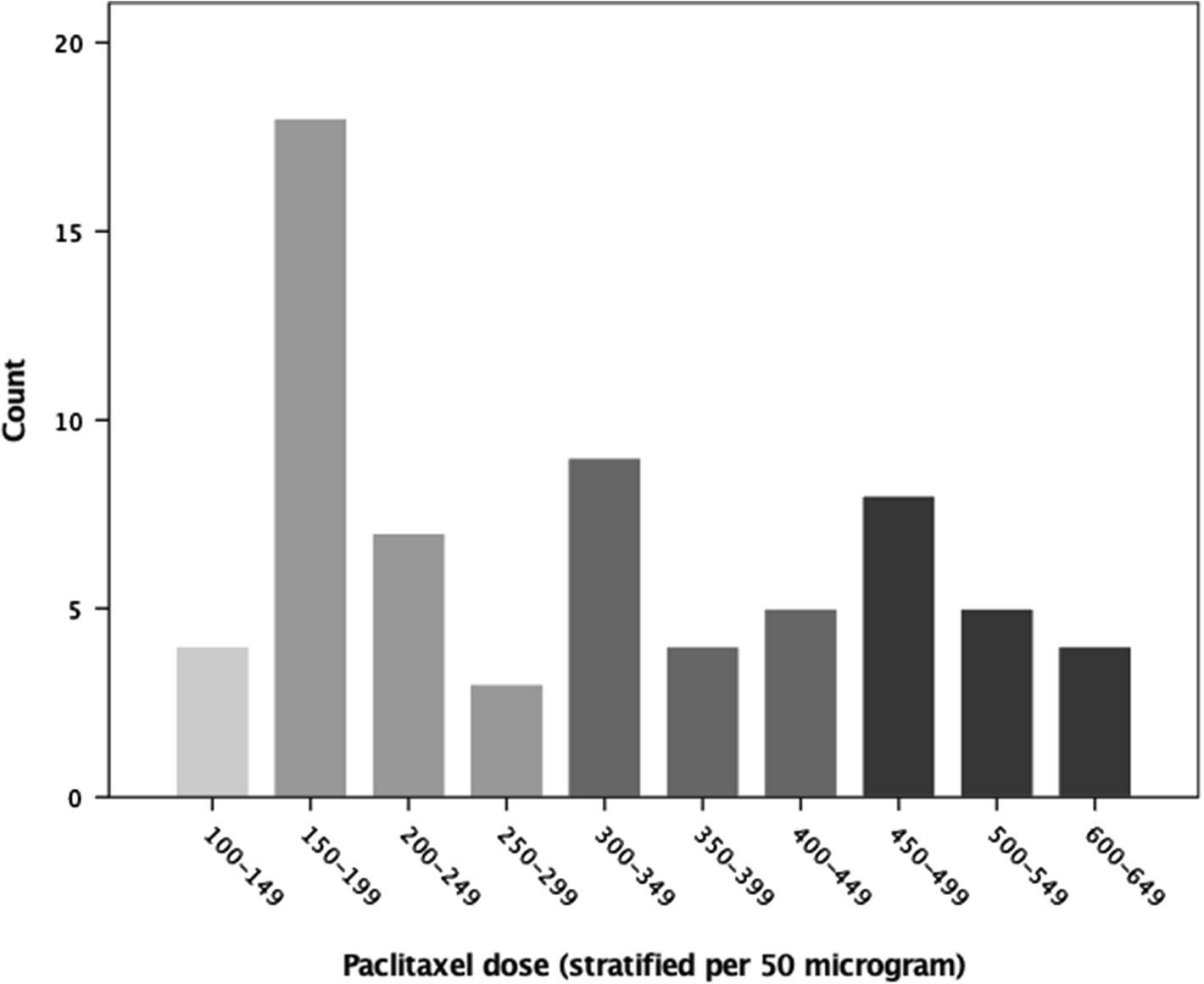
Total paclitaxel dose per patient. The 4 different gray values are corresponding the fourfold classes as used in the analysis in Table 3

The paclitaxel dose in male patients was slightly higher than in female patients (336.61 ± 158.87 vs 273.74 ± 131.15 μcg, respectively). Per added stent the mean, minimum and maximum paclitaxel dose increased. The total paclitaxel dose per patient varied between 116.00 and 645.00 μcg with a mean dose of 315.03 μcg. A paclitaxel dose between 150 and 200 μcg was most frequently observed (see Fig. 2). Patients had a mean body weight of 75.6 ± 12.9 kg. The mean paclitaxel dose per kg was 4.3 μcg with a wide spread (min–max 1.3–11.99 μcg/kg).

### Cox-regression Analysis of Paclitaxel Dose–Response Relationships

No significant univariate hazard ratios were found for the use of paclitaxel-coated DES as a dichotomous variable (HR 1.14, 95% CI 0.78–1.66, *p* = 0.50), for the total dose of paclitaxel as a continuous variable (HR 1.00, 95% CI 0.99–1.00, *p* = 0.99) or for any of the stratified dosages (both univariate or age- and sex-adjusted), or dose-weight ratios (HR 1.05, 95% CI 0.93–1.18, *p* = 0.46), see Table 3. Thus, no significant effects of paclitaxel dose exposure on mortality were observed up to 10 years of analysis.

**Table 3:** 10-year all-cause mortality hazard ratios (unadjusted and adjusted for age and sex) for cumulative paclitaxel dosages

	Univariate			Adjusted for age and sex		
	HR	95% CI	*p *value	HR	95% CI	*p* value
Paclitaxel-DES (dichotomous)	1.14	0.78–1.66	0.50	1.09	0.75–1.58	0.52
Total dose paclitaxel, (continuous) (μcg)	1.0	0.99–1.00	0.90	0.99	0.99–1.00	0.06
Stratified paclitaxel dose (μcg)						
0–149μcg	1.27	0.85–1.87	0.24	1.48	0.86–1.89	0.22
150–299μcg	0.72	0.42–1.21	0.21	0.94	0.55–1.61	0.82
300–449μcg	0.93	0.53–1.65	0.81	0.82	0.46–1.45	0.49
450–700μcg	1.01	0.58–1.78	0.97	0.80	0.45–1.43	0.45
Total dose paclitaxel per weight (continuous) (μcg/kg)	1.05	0.93–1.18	0.46	0.19	0.80–1.05	0.19

Multivariate analyses were carried out to investigate the risk of dose or dose-related effects. The multivariate analysis with all included factors only showed a significant hazard ratio for age (HR 1.08, 95% CI 1.04–1.12, *p *value < 0.005). See Table 4.

**Table 4:** Cox-regression analysis of paclitaxel dose on mortality in patients with paclitaxel-DES

Variables in the equation	HR	95% CI	*p* value
Univariate analysis			
Paclitaxel-DES (dichotomous)	1.14	0.78–1.66	0.50
Multivariate analysis			
Paclitaxel-DES (dichotomous)	1.60	0.19–13.77	0.67
Age (years)	1.08	1.04–1.12	< 0.005
Smoking	1.03	071–1.50	0.89
History of PAD	1.08	0.59–1.98	0.81
Diabetes mellitus	1.48	0.76–2.88	0.24
Previous stroke or transient ischemic attack	1.41	0.67–2.94	0.37
Coronary artery disease	1.47	0.76–2.83	0.25
Anticoagulant medication	1.77	0.63–4.96	0.28

## Discussion

In this present study we investigated the survival of CLI patients treated BTK at 10-year follow-up and the possible influence of treatment with paclitaxel-coated DES.

The main findings of our study are that there is no significant difference between survival in the DES and the PTA ± BMS group at 10-year follow-up. The 10-year survival for both groups was astonishing poor. The second main finding is that no specific paclitaxel-coated DES dose-related mortality and dose per body weight mortality relationships were identified.

Recently, two meta-analyses were published that concluded (1) that there appears to be an increased risk of 5-year mortality of paclitaxel-coated DEB and DES in the superficial femoral and popliteal artery [10] and (2) a significantly worse 1-year amputation-free survival in patients treated with DEB below the knee [11]. These meta-analyses suggested that DEB and DES may be related to poor outcome and should be used with caution.

By focussing on a selected study group within the PADI Trial, we show that this warning does not seem valid for the use of paclitaxel-coated DES in CLI patients treated BTK. We believe there are several explanations for the discrepancies between the PADI Trial and the above-mentioned meta-analyses.

First, we only included patients treated with paclitaxel-coated DES in the PADI Trial, while the meta-analyses mainly included patients treated with DEB. DEB have a substantial higher paclitaxel dose than DES. For example, the 4 × 40 mm IN.PACT DEB (Medtronic, Inc. Minneapolis, United States) [14] and the LUTONIX DEB (Bard Peripheral Vascular, Inc. Tempe, United States) [15] have a 7.21 and a 3.66 times higher dose than the 4 × 38 mm TAXUS Liberté DES (Boston Scientific Corporation, Natick, United States) [13], respectively (see Table 5). In addition, the DES has a scaffold which is not present with DEBs.

**Table 5:** Standardized and commonly used diameters and lengths for both different stents and balloons, so that dose per product could be compared. If the stent diameter or lengths were not available in the product information, the most similar stent was chosen

	Artery	Dose density (μcg/mm^2^)	Length (mm)	Size 1 (small)		Size 2 (large)	
				Diameter (mm)	Dosis (μcg)	Diameter (mm)	Dosis (μcg)
Stents							
TAXUS Liberté (Boston scientific) [13]	BTK, coronaries	1	38	4.0*	273	–	–
ZILVER-PTX (Cook) [20]	Fempop	3	40	5.0	390	7.0	390
Balloons							
LUTONIX (Bard) [15]	Fempop	2	40	4.0	1000	7.0	1800
IN.PACT (Medtronic) [14]	Fempop	3.5	40	4.0	1969	7.0	3819

^*^The TAXUS Liberté DES has a maximum diameter of 4.0 mm, since this stent was originally developed for use in cardiology

Second, femoro-popliteal and BTK arteries differ in diameter. Thus, different stent size and balloon size are used for these vessels. In general, a larger stent or balloon size means a higher paclitaxel dose. However, this increase in dose is less in stents, and larger in balloons. For example, the paclitaxel dose on a TAXUS Liberté stent with a diameter of 2.75 mm and a length of 38 mm is 266μcg which increases to 273μcg (3% dose increase) on a stent with a diameter of 4.0 mm (30% diameter increase) and an equal length. The LUTONIX DEB of 4 × 40 mm has a dose of 1000μcg which increases to a dose of 1800μcg (45% dose increase) on the 6.0 × 40 mm (33% diameter increase).

Third, IC and CLI patients have two different stages of PAD, not only with a different Rutherford category (1, 2, 3 versus 4, 5, 6, respectively), but they also differ significantly in morbidity and survival [16–18].

Finally, there are indications that femoro-popliteal and infrapopliteal/BTK arteries differ in pathology with significantly more atherosclerosis in the SFA, while the BTK arteries have more medial calcifications (arteriosclerosis) and intraluminal thrombi [19].

Thus, as alarming as the results of these meta-analyses are, its results cannot be extrapolated to all subcategories of PAD especially in patients with BTK disease.

### Strengths and Limitations

The major strength of this study is the complete survival analysis up to 10 years after the primary treatment.

This study also has its limitations. The sample size of this study was relatively small and the study design as a post hoc RCT study has its limitations. However, it is the only RCT with such long follow-up. Therefore, a meta-analysis is not possible.

Secondly, for the analysis of the paclitaxel dose, we used the manufacturer's product information. Since we have not performed any checks on the amounts of paclitaxel on the stents, the actual dose in the patient may differ from the doses provided by the manufacturer.

Last, the body weight may have affected the dose body weight analysis. For this study the body weight was used from the electronic patient file. Patients’ weight may fluctuate and especially decrease during follow-up, due to long-term disease burden in this severely affected patient category.

## Conclusions

In conclusion, the 10-year survival of CLI patients treated BTK is poor. There were no significant differences between 10-year mortality in patients with CLI treated BTK with either paclitaxel-coated DES or PTA ± BMS. Despite a relative broad range of paclitaxel per body weight in our patients, no specific dose-related mortality and dose per body weight mortality relationships were identified. Thus, there are no dose-related adverse effects of paclitaxel-coated DES in patients with CLI treated BTK.

## Electronic supplementary material

Below is the link to the electronic supplementary material.

Supplementary file 1 (DOCX 45 kb)
