# Supplementary material for: 10-Year Paclitaxel Dose-Related Outcomes of Drug-Eluting Stents Treated Below the Knee in Patients with Chronic Limb-Threatening Ischemia (The PADI Trial)
Source: Cardiovasc Intervent Radiol. 2020 Jul 28;43(12):1881–8. doi: 10.1007/s00270-020-02602-6 (PMC7649154; doi:10.1007/s00270-020-02602-6)
Supplement: Supplementary file 1 — (DOCX 45 kb) [file 270_2020_2602_MOESM1_ESM.docx]

**SUPPLEMENTAL FIGURES AND TABLES**

Supplemental figure 1. 10-years Kaplan-Meier survival curves for Rutherford classes. Stippled line: Rutherford 4, continuous grey line: Rutherford 5, continuous black line: Rutherford 6.


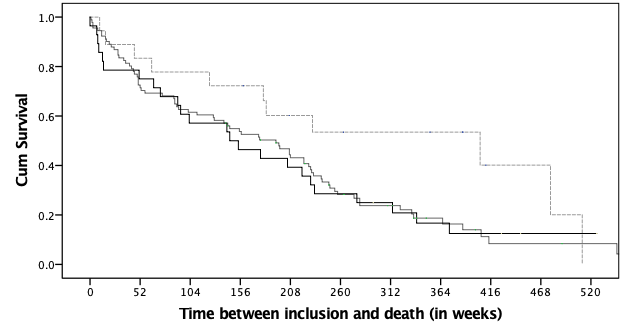


| **Numbers at risk (numbers censored)**  *Log-rank (Mantel-Cox) p-value= 0.13* | | | | | | | | | | | |
| --- | --- | --- | --- | --- | --- | --- | --- | --- | --- | --- | --- |
| **Time(weeks)** | **0** | **52** | **104** | **156** | **208** | **260** | **312** | **364** | **416** | **468** | **520** |
| **Rutherford 4** | 18 (0) | 15 (0) | 14 (0) | 13 (2) | 9 (0) | 8 (1) | 7 (1) | 6 (3) | 2 (0) | 2 (0) | 0 (0) |
| **Rutherford 5** | 91 (0) | 66 (0) | 56 (2) | 47 (2) | 37 (2) | 22 (4) | 15 (4) | 8 (1) | 3 (0) | 3 (1) | 2 (1) |
| **Rutherford 6** | 28 (0) | 21 (0) | 16 (0) | 13 (0) | 11 (0) | 8 (1) | 6 (0) | 4 (0) | 3 (2) | 1 (0) | 1 (1) |

Supplemental figure 2. 10-years Kaplan-Meier survival curves for impaired renal function (eGFR<30 mL/min/1.73m^2^). Light grey line: eGFR<30 mL/min/1.73m^2^, dark grey line: eGFR >30 mL/min/1.73m^2^.


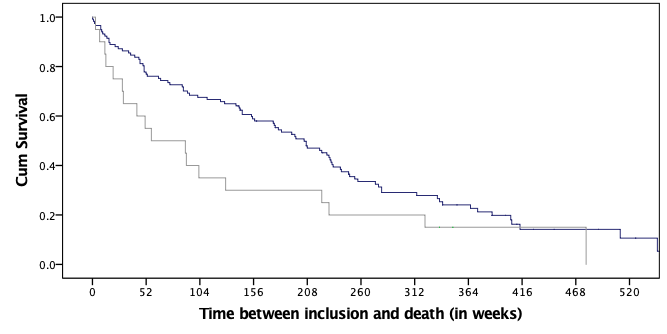


| **Numbers at risk (numbers censored)**  *Log-rank (Mantel-Cox) p-value= 0.04* | | | | | | | | | | | |
| --- | --- | --- | --- | --- | --- | --- | --- | --- | --- | --- | --- |
| **Time(weeks)** | **0** | **52** | **104** | **156** | **208** | **260** | **312** | **364** | **416** | **468** | **520** |
| **eGFR>30** | 117 (0) | 91 (0) | 79 (2) | 67 (4) | 51 (2) | 34 (6) | 24 (3) | 17 (4) | 7 (2) | 5 (1) | 3 (2) |
| **eGFR<30** | 20 (0) | 11 (0) | 7 (0) | 6 (0) | 6 (0) | 4 (0) | 4 (2) | 1 (0) | 1 (0) | 1 (0) | 0 (0) |

Supplemental table 1. Paclitaxel-DES diameters, lengths and associated paclitaxel doses.

| **Nominal expanded stent inner diameter (mm)** | **Nominal unexpanded stent length (mm)** | **Nominal paclitaxel dose (**μ**cg)** |
| --- | --- | --- |
| 2.25 | 32 | 155 |
| 2.50 | 16 | 77 |
| 2.50 | 24 | 116 |
| 2.50 | 28 | 136 |
| 2.50 | 32 | 155 |
| 2.75 | 38 | 266 |
| 3.00 | 24 | 168 |
| 3.00 | 28 | 196 |
| 3.00 | 32 | 224 |
| 3.00 | 38 | 266 |
| 3.50 | 32 | 224 |
| 4.00 | 38 | 273 |

Supplemental table 2. Diameter and length for both DES and BMS. For DES, dose per stent is also shown.

|  | | **DES (n=74)** | | **PTA±BMS (n=66)** | |
| --- | --- | --- | --- | --- | --- |
|  |  | **Mean** ± **SD** | **Min-max** | **Mean** ± **SD** | **Min-max** |
| All stents | Diameter (mm) | 2.83 ± 0.40 | 2.00-4.00 | 3.34 ± 0.53 | 2.50-4.00 |
|  | Length (mm) | 29.74 ± 4.3 | 16-38.00 | 39.17 ± 17.81 | 30-60 |
|  | Paclitaxel dose (μcg) | 179 ± 40 | 77-273.00 | - | - |
| Per patient 3 stents maximum are used. In the control group PTA±BMS all parameters are displayed except dose and dose-related parameters. Data are displayed as mean ± SD, minimum and maximum. | | | | | |
